# Pollen tube contents from failed fertilization contribute to seed coat initiation in *Arabidopsis*

**DOI:** 10.12688/f1000research.18644.2

**Published:** 2019-04-30

**Authors:** Xiaoyan Liu, Parakash Babu Adhikari, Ryushiro D. Kasahara

**Affiliations:** 1 School of Life Sciences, Fujian Agriculture and Forestry University, Fuzhou, Fujian, 350002, China; 2 FAFU-UCR Joint Center and Fujian Provincial Key Laboratory of Haixia Applied Plant Systems Biology, Haixia Institute of Science and Technology, Fujian Agriculture and Forestry University, Fuzhou, Fujian, 350002, China

**Keywords:** Arabidopsis, Pollen tube, fertilization recovery system, pollen tube-dependent ovule enlargement morphology, GCS1, pollen tube contents, Seed coat initiation, AGL62

## Abstract

Plant seeds are essential for human beings, constituting 70% of carbohydrate resources worldwide; examples include rice, wheat, and corn. In angiosperms, fertilization of the egg by a sperm cell is required for seed formation; therefore, fertilization failure results in no seed formation, except in the special case of apomixis. Initially, plants produce many pollen grains inside the anthers; once the pollen grain is deposited onto the top of the pistil, the pollen tube elongates until it reaches the ovule. Generally, only one pollen tube is inserted into the ovule; however, we previously found that if fertilization by the first pollen tube fails, a second pollen tube could rescue fertilization via the so-called fertilization recovery system (FRS). Our previous reports also demonstrated that failed fertilization results in pollen tube-dependent ovule enlargement morphology (POEM), enlarged seeds, and partial seed coat formation if the pollen tube releases the pollen tube contents into the ovule. However, we have not determined whether all the ovules enlarge or produce seed coats if an ovule accepts the pollen tube contents. Therefore, we conducted a partial seed coat formation experiment taking into account both the FRS and POEM phenomena. Notably, the ratios of failed fertilization and the ovules with partial seed coats matched, indicating that all ovules initiate seed coat formation if the fertilization fails but the pollen tube contents enter the ovule. In addition, we confirmed that the *agl62* mutant , defective in early endosperm formation, showed seed coat initiation with and without fertilization, indicating that for a normal seed coat initiation, fertilization is not required; however, for the completion of normal seed coat formation, both normal fertilization and endosperm formation are required. Further molecular evidence is required to understand these phenomena because very few factors related to FRS and POEM have been identified.

## Introduction

In angiosperms, seed formation begins with pollination ^1, 2^. Once the pollen grain lands on the stigma at the top of the pistil, pollen tubes from the grain elongate toward the ovule. Fusion of the two gametes is required for seed formation. The male gametophyte is the pollen grain and the female gametophyte is the embryo sac ^3^. Immediately after arrival at the ovules, the pollen tube bursts and the pollen tube contents (PTC) are released to the female gametophyte ^4^.

In a previous study, we reported that once the ovule accepts the PTC inside the female gametophyte, it begins enlargement and seed coat formation, irrespective of fertilization ^5, 6^. We named this phenomenon pollen tube-dependent ovule enlargement morphology (POEM). We also reported that if fertilization of the ovule fails, a partial seed coat is still produced, even though a complete seed coat cannot be formed. However, we have not confirmed whether all the ovules have the partial seed coat phenotype when ovule fertilization fails but PTC is accepted. To address this question, statistical experiments were conducted that included the fertilization recovery system (FRS), where a second pollen tube rescues the fertilization if fertilization by the first pollen tube fails, which we previously identified ^7, 8^. We reported that the seed formation ratio of *gcs1*/+ mutants ^9– 11^ was approximately 65%; the remaining mutants were unable to produce seeds because fertilization of these ovules failed. Therefore, matching of the ratio of the ovules with the partial seed coat phenotype to the seed abortion ratio suggests that there was fertilization failure for all ovules with a partial seed coat. We also conducted experiments to determine whether *agl62* mutants ^12^ had the partial seed coat phenotype. In *agl62* seeds, the endosperm cellularizes prematurely, indicating that AGL62 is required for suppression of cellularization during the syncytial phase. During seed development, *AGL62* is exclusively expressed in the endosperm. Because *agl62* mutants have an abnormal endosperm phenotype after central cell fertilization, these mutants are ideal for investigating the relationship between endosperm formation and seed coat initiation and formation.

## Methods

### Plant materials and growth conditions

*Arabidopsis thaliana* ecotype Columbia (Col-0) plants were used as the wild-type (WT) plants. Test cross experiments were conducted in *gcs1*/+ ^9– 11^, *agl62*/+ ^12^, and WT plants. Seeds were sterilized with 5% sodium hypochlorite containing 0.5% Triton X-100 and germinated on plates containing 0.5× Murashige and Skoog salts (pH 5.7) (Wako Pure Chemical), 2% sucrose, Gamborg’s B5 vitamin solution (Sigma), and 0.3% Gelrite (Wako Pure Chemical) in a growth chamber at 21.5°C under 24 h of light after cold treatment (4°C) for 2 days. Next, 10-day-old seedlings were transferred to Metro-Mix 350 soil (Sun Gro) and grown at 21.5°C under 24 h of light.

### Phenotypic analyses

For staining the silique tissue, the WT flowers were emasculated at stage 12c ^13^ and pollinated with *gcs1*/+ pollen grains. For *agl62* experiments, the *agl62* mutant flowers were emasculated at stage 12c and pollinated with WT, *gcs1*/+, and *agl62* pollen grains. The siliques were collected at 3 days after pollination (DAP).

For vanillin staining, the ovules were manually dissected from the ovaries and mounted on slides in 1% (wt/vol) vanillin (4-hydroxy-3-methoxybenzaldehyde; Sigma) in 6 N HCl solution. Slides were analyzed after 20 min of incubation. Samples were analyzed with a Leica DM2500 microscope using differential interference contrast optics. Images were recorded with a Leica DFC 300 FX digital camera at a magnification of 5×, 10× and 20×. The microscopic protocols followed were as previously described ^5^.

## Results and discussion

**Figure 1.**
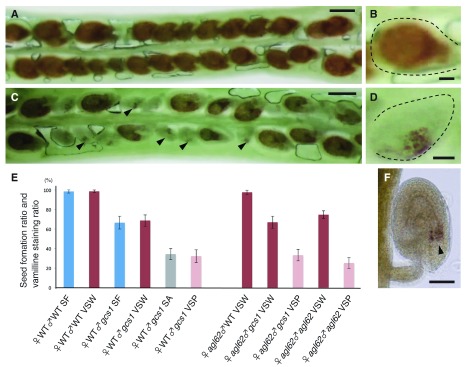
Pollen tube content (PTC) is sufficient to initiate seed coat formation. ( **A**) Wild-type (WT) silique crossed with WT pollen and stained with vanillin. Almost all ovules were stained. Bar: 300µm. ( **B**) Representative image of whole seed coat staining. Bar: 50µm. ( **C**) WT silique crossed with *gcs1* pollen and stained with vanillin. Several ovules had partial seed coat (arrowhead) staining. Bar: 300µm. ( **D**) Representative image of partial seed coat staining. Bar: 50 µm. ( **E**) Comparison of seed formation ratio and vanillin staining ratio. ♀WT♂WT seed formation ratio (SF) indicates that a WT silique was crossed with WT pollen and the seed formation ratio was calculated. ♀WT♂WT vanillin stained in whole seed coat (VSW) indicates that a WT silique was crossed with WT pollen and the whole seed coat staining ratio was calculated. ♀ *agl62*♂WT VSW indicates that an *agl62*/+ silique was crossed with WT pollen and the whole seed coat staining ratio was calculated. ♀WT♂ *gcs1* SF indicates that a WT silique was crossed with *gcs1*/+ pollen and the seed formation ratio was calculated. ♀WT♂ *gcs1* VSW indicates that a WT silique was crossed with *gcs1*/+ pollen and the whole seed coat staining ratio was calculated. ♀ *agl62*♂ *gcs1* VSW indicates that an *agl62*/+ silique was crossed with *gcs1*/+ pollen and the whole seed coat staining ratio was calculated. ♀WT♂ *gcs1* seed abortion ratio (SA) indicates that a WT silique was crossed by *gcs1*/+ pollen and the seed abortion ratio was calculated. ♀WT♂ *gcs1* (vanillin stained in partial seed coat) indicates that a WT silique was crossed with *gcs1*/+ pollen and the partial seed coat staining ratio was calculated. ♀ *agl62*♂ *gcs1* VSP indicates that an *agl62*/+ silique was crossed with *gcs1*/+ pollen and the partial seed coat staining ratio was calculated. ♀ *agl62*♂ *agl62* VSW indicates that an *agl62*/+ silique was crossed with *agl62*/+ pollen and the whole seed coat staining ratio was calculated. ♀ *agl62*♂ *agl62* VSP indicates that an *agl62*/+ silique was crossed with *agl62/+* pollen and the partial seed coat staining ratio was calculated. Blue bars indicate SF (Seed formation ratio). Dark red bars indicate VSW (vanillin stained in whole seed coat). Gray bar indicates SA (Seed abortion ratio). Pink bars indicate VSP (vanillin stained in partial seed coat). ( **F**) A ♀ *agl62*♂ *agl62* VSP ovule. The arrowhead indicates the vanillin-stained zone. Bar: 50 µm. For normal seed coat initiation, fertilization is not required; however, for completion of normal seed coat formation, both normal fertilization and normal endosperm development are required.

First, the WT plants as both the female and male parent ( Figure 1A) were crossed and the silique after vanillin staining at 3DAP was observed. The ratio of full seed coat formation was 98.7±1.2% (mean ± SD; n=10 pistils), which was consistent with our previous WT fertilization data ^7, 8^. By contrast, when the WT plants as the female parent and *gcs1*/+ as the male parent were crossed, the ratio of full seed coat formation was 68.7±5.8% (n=10 pistils) and the ratio of partial seed coat formation was 32.2±6.5% (n=10 pistils), which also was consistent with our previous *gcs1*/+ fertilization data. These data suggest that all successfully fertilized ovules produce a full seed coat and all unfertilized but PTC accepted ovules produce a partial seed coat.

Because the *agl62* mutant had an abnormal and arrested endosperm formation phenotype after fertilization, this mutant was ideal for investigating the relationship between endosperm formation and seed coat initiation and formation. The *agl62/+* plants as the female parent and the WT as the male parent were crossed ( Figure 1) and the silique after vanillin staining at 3DAP was observed. The ratio of full seed coat formation was 97.6±2.1% (n=10 pistils), which was consistent with our previous WT fertilization data. By contrast, when *agl62/+* plants as the female parent and *agl62/+* as the male parent were crossed, the ratio of full seed coat formation was 74.7±3.9% (n=10 pistils) and the ratio of partial seed coat formation was 25.2±5.4% (n=10 pistils), which was consistent with previous *agl62* data ^12^. These results suggest that normal endosperm development is required for completion of seed coat formation, irrespective of fertilization. When *agl62/+* plants as the female parent and *gcs1*/+ as the male parent were crossed, the ratio of full seed coat formation was 66.9±6.2% (n=10 pistils) and the ratio of partial seed coat formation was 33.2±5.9% (n=10 pistils), which also was consistent with our previous *gcs1*/+ fertilization data. These results suggest that *agl62*/+ abnormal endosperm prevents normal seed coat formation, but these ovules still produce a partial seed coat because these ovules had accepted the PTC. In summary, for normal seed coat initiation, fertilization is not required; however, for completion of normal seed coat formation, both normal fertilization and normal endosperm development are required.

## Data availability

Open Science Framework: Vanillin staining project. https://doi.org/10.17605/OSF.IO/6U73H ^14^.

This project contains the following underlying data:

- # of seeds data (Sheet2 contains the number of seeds stained out of the total number of seeds; Sheet1 the data summary used to produce Figure 1E)
- agl62-3v.tif (raw image of stained *agl62*/+ seeds)
- gcs1 vaniline.tif (raw image of stained *gcs1*/+ seeds)
- WT vaniline.tif (raw image of stained wild-type seeds)

Data are available under the terms of the Creative Commons Zero “No rights reserved” data waiver (CC0 1.0 Public domain dedication).
