# Supplementary material for: Pollen tube contents from failed fertilization contribute to seed coat initiation in Arabidopsis
Source: F1000Res. 2019 Apr 30;8:348. Originally published 2019 Mar 28. [Version 2] doi: 10.12688/f1000research.18644.2 (PMC6468697; doi:10.12688/f1000research.18644.2)
Supplement: Peer review history

# Peer review: Reviewer response for version 2

**DOI:** 10.5256/f1000research.20759.r47869

**Published:** 2019-05-07

**Authors:** Tomoko Igawa

**Affiliations:** 1 Graduate School of Horticulture, Chiba University, Chiba, Japan

The author revisions are sufficient for the comments by the reviewers. The revised paper is acceptable for indexing.

I have read this submission. I believe that I have an appropriate level of expertise to confirm that it is of an acceptable scientific standard.

# Peer review: Reviewer response for version 1

**DOI:** 10.5256/f1000research.20411.r46516

**Published:** 2019-04-15

**Authors:** Tomokazu Kawashima

**Affiliations:** 1 Department of Plant and Soil Sciences, University of Kentucky, Kentucky, USA

This paper describes that the pollen tube content is sufficient to partially initiate seed coat without fertilization in Arabidopsis. This work appears the extension and further clarification of the previously published work (Kasahara et al., Science Advances 2016 ^1^, and Roszak and Kohler, PNAS, 2011 ^2^). During plant fertilization, many cellular processes are happening, and it is important to integrate the identified phenomena together for comprehensive understanding of plant sexual reproduction. The authors studied the relationships among FRS, POEM, seed coat initiation, and endosperm development, and I think the work reported in this paper is an important piece of information in sexual plant reproduction.

 I have a few suggestions:

1. The first paragraph of the introduction, the author stated that "gametophytes" fuse. I believe the authors mean "gametes".
2. From the authors previous work in Science Advances, 2016 ^1^), it is clear that PTC initiates seed coat development (transcriptionally and anatomically). It is important to state these details in the introduction to remind the readers. Along with this, please describe what vanillin staining detects (proanthocyanidin accumulation) and why this can be used for seed coat visualization.
3. In Roszak and Kohler, 2011 ^2^, the authors reported that not *fis2* and *mea*, but *fie* and *msi1* autonomously developing seeds differentiate seed coat, suggesting that fertilization is not required for seed coat initiation. The authors also described seed coat phenotypes in *agl62*. Please refer this publication and highlight and/or discuss new findings/hypotheses in this paper, then this paper will be more impactful.
4. As other reviewers suggested, Figure 1E is difficult to dissect information. I believe "vamiline" in the Y-axis label should be "vanillin".

I have read this submission. I believe that I have an appropriate level of expertise to confirm that it is of an acceptable scientific standard.

## References

[1] KasaharaRDNotaguchiMNagaharaSSuzukiTSusakiDHonmaYMaruyamaDHigashiyamaT: Pollen tube contents initiate ovule enlargement and enhance seed coat development without fertilization. *Sci Adv*.2016;2(10) : 10.1126/sciadv.1600554 e1600554 10.1126/sciadv.1600554 27819041PMC5091356

[2] RoszakPKöhlerC: Polycomb group proteins are required to couple seed coat initiation to fertilization. *Proc Natl Acad Sci U S A*.2011;108(51) : 10.1073/pnas.1117111108 20826-31 10.1073/pnas.1117111108 22143805PMC3251106

# Response

**Published:** 2019-04-15

**Authors:** Kasahara Ryushiro

**Affiliations:** Fujian Agriculture and Forestry University, China

Dear Dr. Tomokazu Kawashima,

 Thank you very much for your critical comments.

 We will improve our paper based on your suggestion.

 Again, thank you very much.

 Ryushiro Dora Kasahara

# Response

**Published:** 2019-04-15

**Authors:** Kasahara Ryushiro

**Affiliations:** Fujian Agriculture and Forestry University, China

Dear Dr. Tomokazu Kawashima,

Thank you very much for your critical comments.

We will improve our paper based on your suggestion.

Again, thank you very much.

Ryushiro Dora Kasahara

# Peer review: Reviewer response for version 1

**DOI:** 10.5256/f1000research.20411.r47023

**Published:** 2019-04-11

**Authors:** Nobutaka Mitsuda

**Affiliations:** 1 Bioproduction Research Institute, National Institute of Advanced Industrial Science and Technology (AIST), Tsukuba, Japan

This paper showed basically the confirmation of the previous report of the POEM paper. However, because this report showed that the relationships between pollen tube contents and seed coat formation statistically, including the data for agl62 mutants, they clearly confirmed that almost all ovules started seed coat formation without fertilization but just required for the pollen tube contents. I believe this report is a sort of important discoveries in the plant science field and will contribute to further research for seed coat formation in angiosperms.

 Introduction:

- At the introduction part, it is clear what they need to understand and what sorts of approach were taken. I believe their approach conducted here is adequate and leads to a neat statistics for seed coat initiation and completion study.  

Results and Discussion:

- I agree with the first reviewer’s comment. Authors could improve the paper if they change the colors for Fig. 1. However, I think this change is not mandatory because if authors change the color dramatically, it could be too showy. I thought current style looks fair enough.
- I also agree with the first reviewer's comment that because the study of agl62 was not the authors study, "our previous agl62 data" sounds strange. But this is just a very minor correction to be done.

Data availability:

- I think the data title agl61-3v. tif is wrong. Title should be "agl62-3v".

I have read this submission. I believe that I have an appropriate level of expertise to confirm that it is of an acceptable scientific standard.

# Response

**Published:** 2019-04-11

**Authors:** Kasahara Ryushiro

**Affiliations:** Fujian Agriculture and Forestry University, China

Dear Dr. Mitsuda,

Thank you very much for your critical comments.

We will improve our paper based on your suggestion.

Again, thank you very much.

Ryushiro Dora Kasahara

# Peer review: Reviewer response for version 1

**DOI:** 10.5256/f1000research.20411.r46381

**Published:** 2019-04-08

**Authors:** Tomoko Igawa

**Affiliations:** 1 Graduate School of Horticulture, Chiba University, Chiba, Japan

Normal seed formation required for the birth of the progeny is a critical phenomenon for plant production and breeding field.

 This research note reports that the involvements of the fertilization and the normal endosperm development in seed coat formation. First, the involvement of the pollen tube contents was analyzed showing the reproducibility of their previous report. Also, it was indicated that normal endosperm development is required for successive seed coat formation, using agl62 mutant.

**Introduction**

- Authors described constructively what they wanted to clarify. The experimental design and the statistical analysis performed in this study were enough to examine the questions.

  
**Results and Discussion**

- In the present state, the descriptions and the graph (Fig.1E) were confusing to understand the results. How about using different colors for each SF, VSF, AS, and VSP. Also, if the graph-bars obtained with the same female parent-genotype are grouped and indicated, it would be helpful for easy comparing the results.
- As a conclusion, the authors claimed that "for completion of normal seed formation, normal endosperm formation is required in addition to normal fertilization." In the previous studies, the aborted seed caused by the single fertilization of the central cell seemed to have developed the seed coat (kokopelli, gex2, dmp9 mutants, etc...). These studies are supportive of the authors' claim, but the endosperm of these mutants was not normal as the wild type seed. Therefore, how about describing "normal endosperm 'development' is required for seed coat formation."
- As a minor point, authors describe the previous study of agl62 (Kang et al., 2008) ^1^ as 'our previous....' but no same authors in this paper.

Overall, the content of this research note is logically written. If the points described above are re-considered, it would be improved.

I have read this submission. I believe that I have an appropriate level of expertise to confirm that it is of an acceptable scientific standard, however I have significant reservations, as outlined above.

## References

[1] KangIHSteffenJGPortereikoMFLloydADrewsGN: The AGL62 MADS domain protein regulates cellularization during endosperm development in Arabidopsis. *Plant Cell*.2008;20(3) : 10.1105/tpc.107.055137 635-47 10.1105/tpc.107.055137 18334668PMC2329934

# Response

**Published:** 2019-04-08

**Authors:** Kasahara Ryushiro

**Affiliations:** Fujian Agriculture and Forestry University, China

Dear Dr. Igawa,

 Thank you very much for your critical comments. I will improve the paper based on your comments as soon as possible.

 Best regard,

 Ryushiro Dora Kasahara

# Response

**Published:** 2019-04-11

**Authors:** Kasahara Ryushiro

**Affiliations:** Fujian Agriculture and Forestry University, China

Dear Dr. Igawa,

I have finished revising based on your suggestions.

In the present state, the descriptions and the graph (Fig.1E) were confusing to understand the results. How about using different colors for each SF, VSF, AS, and VSP. Also, if the graph-bars obtained with the same female parent-genotype are grouped and indicated, it would be helpful for easy comparing the results.

I have submitted a new version of the Fig.1. Please take a look.

As a conclusion, the authors claimed that "for completion of normal seed formation, normal endosperm formation is required in addition to normal fertilization." In the previous studies, the aborted seed caused by the single fertilization of the central cell seemed to have developed the seed coat (kokopelli, gex2, dmp9 mutants, etc...). These studies are supportive of the authors' claim, but the endosperm of these mutants was not normal as the wild type seed. Therefore, how about describing "normal endosperm 'development' is required for seed coat formation."

I changed the word to normal endosperm development.

As a minor point, authors describe the previous study of agl62 (Kang et al., 2008) ^1^ as 'our previous....' but no same authors in this paper.

I removed "our". from the text.

Again, thank you very much for your critical comments.

Best regards,

Ryushiro Dora Kasahara
